# Convalescent serum therapy for COVID-19: A 19th century remedy for a 21st century disease

**DOI:** 10.1371/journal.ppat.1008735

**Published:** 2020-08-12

**Authors:** Daniel Montelongo-Jauregui, Taissa Vila, Ahmed S. Sultan, Mary Ann Jabra-Rizk

**Affiliations:** 1 Department of Oncology and Diagnostic Sciences, School of Dentistry, University of Maryland, Baltimore, Maryland, United States of America; 2 Department of Microbiology and Immunology, School of Medicine, University of Maryland, Baltimore, Maryland, United States of America; University of Kentucky, UNITED STATES

## Bridging the gap between now and then

Coronavirus disease 2019 (COVID-19) is a viral respiratory disease that mysteriously emerged in late December 2019 in Wuhan City, China [1, 2]. The virus quickly spread worldwide and was announced a global pandemic by the World Health Organization (WHO) in March 2020. Shortly after, a novel coronavirus was identified as the etiologic agent and named severe acute respiratory syndrome coronavirus 2 (SARS-CoV-2), the seventh coronavirus to infect humans and the third to cause an outbreak [2–4]. SARS-CoV-2 continues to spread around the world, as of late July, 2020, over 17 million people have been infected, causing over 665,000 deaths (https://coronavirus.jhu.edu/) and these numbers continue to trend upwards. Despite concerted global efforts, only a few targeted therapeutics, such as remdesivir, are available to help prevent or treat this disease [5], therefore, convalescent plasma therapy, a century-old medical remedy is being revisited as a viable and immediate option for mitigating the impact of this disease [6]. Convalescent plasma therapy is a type of passive antibody therapy whereby blood plasma with neutralizing antibodies against a specific virus is recovered from people who have recuperated from an infection, and administered to patients with the infection in order to improve clinical outcome [6]. Although the potential clinical benefit of convalescent plasma therapy in COVID-19 is still uncertain, administering antibody-containing plasma from recovered patients is a near-term option that can be implemented relatively quickly. In fact, because of the high number of patients with severe COVID-19 and the mainstay of current clinical treatment consisting of symptomatic management and mechanical ventilation, administering convalescent plasma for treatment purposes is currently being deployed [7–12]. Although it is still early to tell whether this therapeutic approach is effective against this disease, evidence so far has shown promise in critically ill patients [7–10]. As new targeted therapies against COVID-19 take considerable time to develop, test and deploy, convalescent plasma therapy could buy time needed to develop more sophisticated targeted treatments.

## Historical precedent for the use of antibody therapy

**Fig 1:**
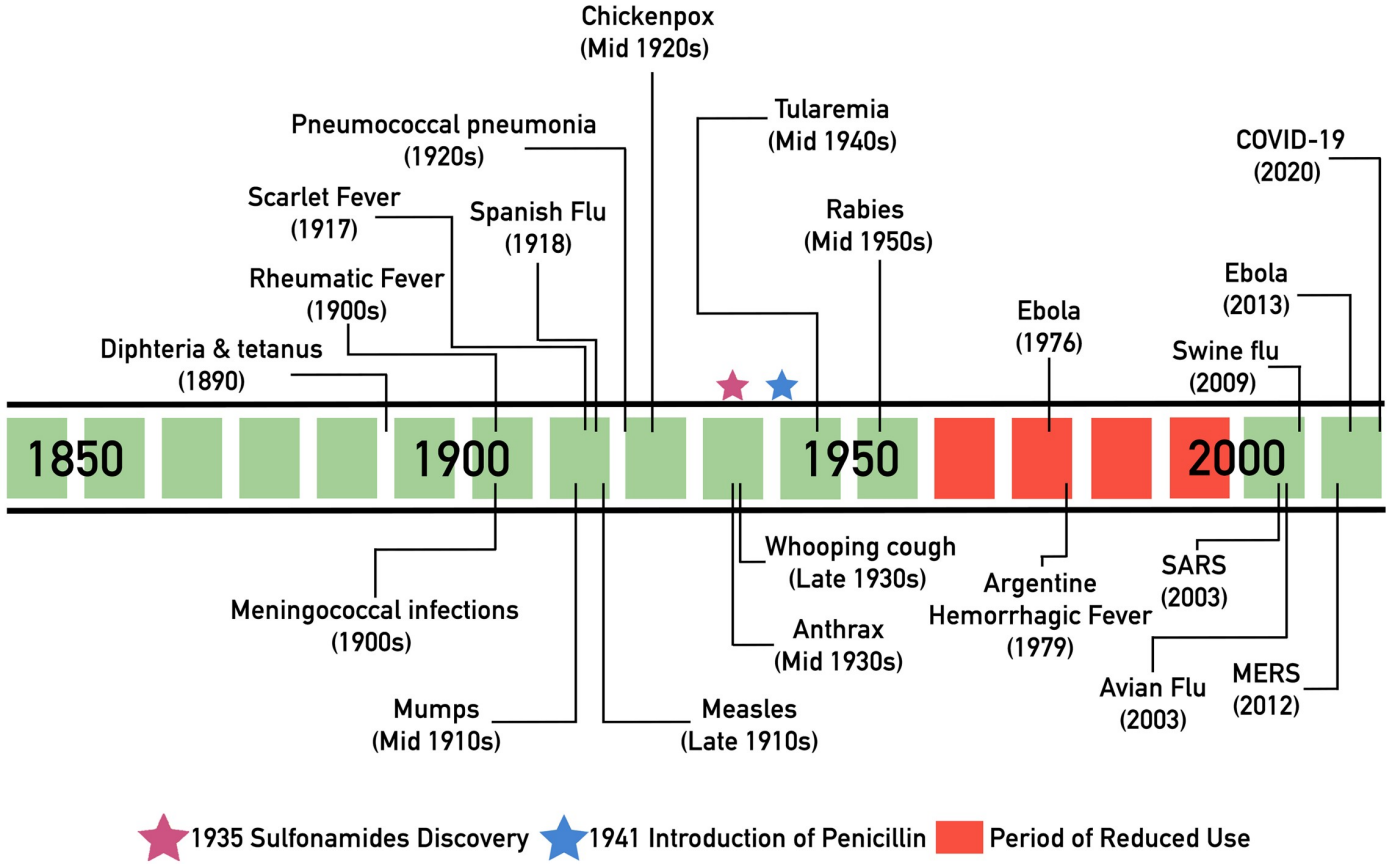
Notable historic uses of antibody therapy against infectious diseases. COVID-19, coronavirus disease 2019; MERS, Middle East respiratory syndrome; SARS, severe acute respiratory syndrome.

Prior to the antibiotic era, serum (plasma minus clotting factors) therapy was widely used to treat a range of infectious diseases such as scarlet fever and pneumococcal pneumonia. In 1890, the physiologists von Behring and Kitasato used blood serum from immunized animals to treat diphtheria and tetanus [13]; subsequently, serum from recovered animals was identified as a possible source of specific antibodies [14, 15]. The use of convalescent serum gained global recognition and revolutionized the way infectious diseases were treated, and in 1901, Emil von Behring was awarded the Nobel Prize for Medicine for his work, which served as a basis for treatment of multiple diseases in the 1900s as well as the development of vaccines [15]. In fact, there are numerous examples throughout history in which convalescent serum was used with some degree of success to treat an array of diseases, including rheumatic fever [16], scarlet fever [17], mumps [18], measles [18, 19], chickenpox [18], and pneumococcal and meningococcal infections [20] (Fig 1). Most notable use was during the Spanish Flu pandemic (1918 to 1920), where meta-analysis studies showed a significantly reduced mortality risk in patients treated with convalescent serum [8, 12]. However, with the advent of antimicrobials, by the middle of the 20th century, the use of serum therapy had declined. Nevertheless, the interest in passive antibody therapy has been renewed periodically when new epidemics or pandemics have emerged. One example is during the Ebola virus (EBOV) outbreak in 1976 in the Democratic Republic of Congo, where an infected laboratory worker recovered after transfusion with convalescent plasma containing anti-EBOV antibodies. Similarly, in 1979, patients with Argentine hemorrhagic fever virus treated with convalescent plasma had a lower mortality rate compared with subjects treated with normal plasma, and similar results were reported for subsequent epidemics of the disease [21]. Over the following decades, convalescent plasma therapy was successfully employed during the H1N1 swine influenza pandemic (2009), the H5N1 avian flu epidemic (2003), as well as during the EBOV outbreak in West Africa in 2013. Most relevant and encouraging is the use of convalescent plasma during 2 previous coronavirus epidemics: severe acute respiratory syndrome (SARS) in 2003, and Middle East respiratory syndrome (MERS) in 2012 [21]. The high degree of success in achieving favorable clinical outcomes during these coronaviruses outbreaks establishes a strong precedent and supports the notion that convalescent plasma could be a viable option for treatment of COVID-19 patients, particularly upon early administration [6, 9, 12, 21–23].

## Buying time with the help of the convalescent

The convalescent plasma therapeutic approach is based on the principle of passive antibody therapy, a short-term strategy whereby antibodies from the blood of someone who recovered from an infection can be administered to protect or treat another person [6, 21]. Effectively, the end goal is the same as vaccines, making antibodies against a specific infectious agent readily available. For instance, a vaccine relies on the host immune cells (B lymphocytes specifically) to produce antibodies after antigen recognition and signal amplification by the immune system, a process that may take weeks [24]; on the other hand, in the case of passive antibody therapy, the process is expedited by providing a patient with immediate immunity when the premade antibodies are given. Therefore, for COVID-19 patients, the expedited approach could prove lifesaving. Nevertheless, this advantage does not come without caveats, as immunization with passive antibody therapy is typically of shorter-term protection, in part because of the half-life of antibodies in circulation [25] and lack of new production by B lymphocytes. Today, passive antibody therapy relies primarily on pooled immunoglobulin preparations that contain high concentrations of antibodies. In contrast, plasma has been used emergently in epidemics in which there is insufficient time or resources to generate immunoglobulin preparations [21].

**Fig 2:**
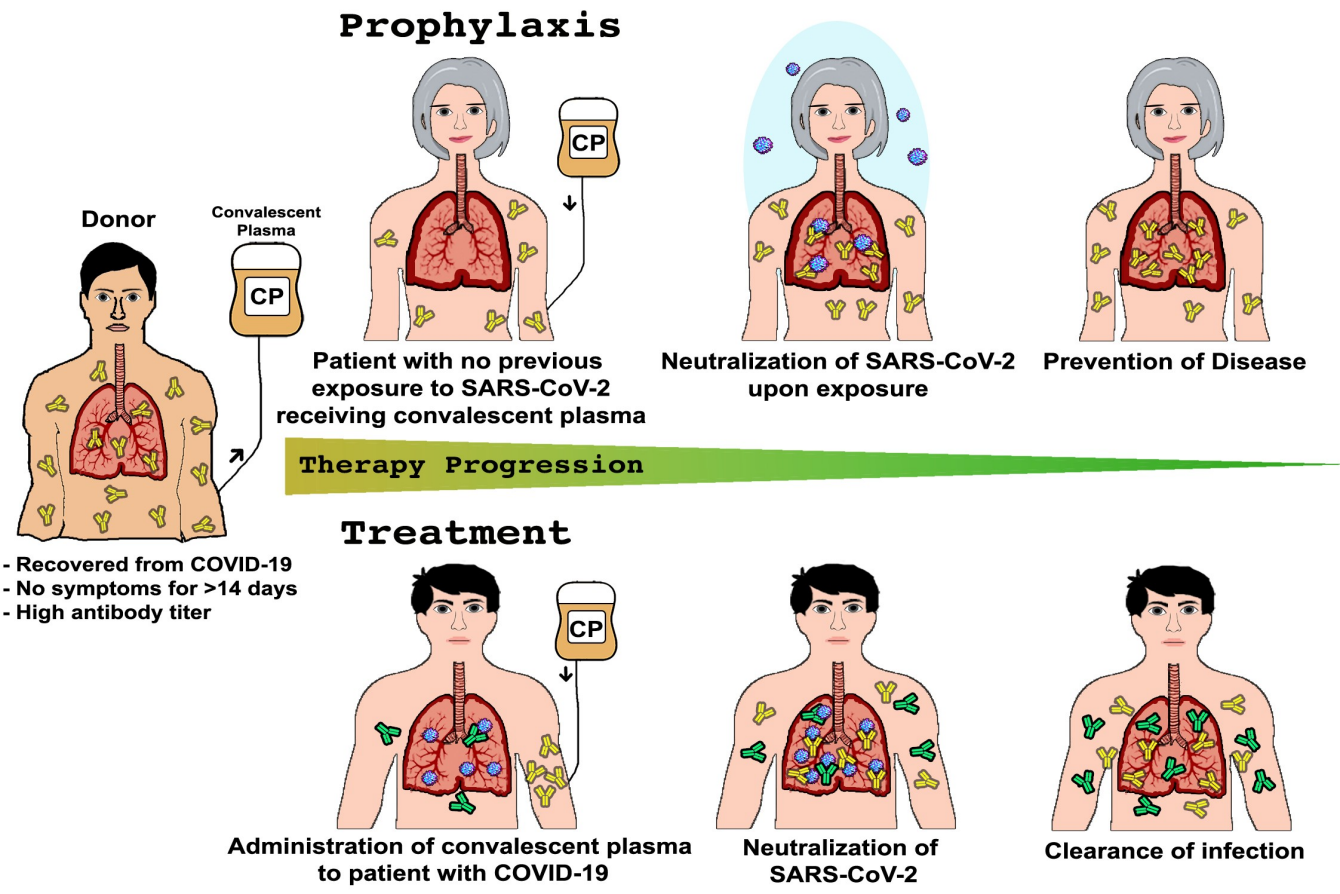
Overview of the use and applications of CP therapy. Virus-neutralizing antibodies in the plasma of a patient who recovered from COVID-19 can be administered prophylactically to prevent infection in vulnerable individuals and those with known exposure to the virus (Prophylaxis). Convalescent plasma can also be administered to infected individuals to improve the clinical outcome (Treatment). CP, convalescent plasma; COVID-19, coronavirus disease 2019; SARS-CoV-2, severe acute respiratory syndrome coronavirus 2.

Despite the high rate of SARS-CoV-2 infection, the relatively low mortality rate provides a rich pool of donors [26]. However, potential COVID-19 donors must meet several eligibility criteria that ensure the donor has antibodies against SARS-CoV-2 and lacks the presence of other types of infections [21, 27]. Additionally, only plasma with high anti-SARS-CoV-2 titers of immunoglobulins G and M (IgG and IgM) are used. Once collected, plasma can be tested and administered within hours, following conventional donor–patient blood compatibility typing [27]. In addition to rapid mobilization, this therapeutic approach is also versatile in applicability as it can be used for prophylaxis or treatment, as illustrated in Fig 2. In the case of prophylaxis, a subject considered at high risk for infection (because of age or underlying medical conditions or who is likely to be in contact with people with COVID-19) could be administered convalescent plasma or neutralizing antibodies for protection against infection. Alternatively, plasma can be administered to treat subjects who have contracted the infection but have not made sufficient antibodies against the virus yet in order to augment their immune response, improve disease course, and enhance recovery [21]. However, it is important to note that passive antibody therapy is most effective when administered prophylactically or implemented early after the onset of symptoms [21].

Convalescent plasma with neutralizing antibodies is currently being used for investigational purposes in the COVID-19 pandemic, and preliminary results from 2 small studies performed in China are encouraging. A pilot study exploring the feasibility of convalescent plasma transfusion to rescue a group of 10 patients with severe disease showed that 1 dose (200 mL) of convalescent plasma with high neutralizing antibody titers was well tolerated, resulted in disappearance of viremia, and improved clinical symptoms in all patients within days of administration [8]. Similar results were reported from another study with 5 critically ill patients on mechanical ventilation [7]. Although these small, nonrandomized studies had limitations, these findings indicate that convalescent plasma could be a promising rescue option for severe COVID-19 [21].

## Limitations and potential risks

Although convalescent plasma therapy is considered a relatively safe therapeutic modality, there are some potential risks [12]. One theoretical complication that may arise is an antibody-mediated proinflammatory disease enhancement known as antibody-dependent enhancement (ADE), whereby antibodies that developed during a prior infection exacerbate severity of the disease [12, 28]. The transfer of these antibodies may aberrantly activate fragment crystallizable (Fc) or complement receptors, increasing recruitment of proinflammatory cytokines and chemokines to the site of infection and causing severe tissue damage [29–31]. Additionally, the presence of non-neutralizing antibodies may exacerbate viral endocytosis or phagocytosis into host cells via Fc receptors, potentializing viral replication [29, 32]. However, although this phenomenon is well known with Dengue and other viral diseases, there have not been any reported ADE cases with the use of convalescent plasma for SARS, MERS, or COVID-19 [12, 29, 32–35]. Nevertheless, it is crucial to have a clear understanding of the role of the recipients' immune response [12]. Transmission of the virus through transfusion is another concern; however, the risk is relatively low because of strict transfusion protocols, and when used for treatment purposes, the recipient is already infected [12, 36].

The success of convalescent plasma therapy hinges on the availability of plasma with high concentrations of antibodies, which may not be a major limitation for COVID-19 because SARS-CoV-2 has been shown to elicit high neutralizing antibody titers in recently convalesced individuals [7–9]. Apheresis is an automated technology that allows for selective collection of a blood fraction while other components can be transfused back to the donor. Therefore, for donation of convalescent plasma, plasmapheresis is recommended because it is highly efficient and approximately 400–800 mL of plasma can be collected in a single donation, providing 2–4 units of convalescent plasma for transfusion [21]. There are some practical and logistical limitations for the implementation of a large-scale convalescent plasma transfusion program such as training of study personnel, recruitment of donors, and transport of plasma to hospitals, as well considerations for plasma shelf-life and half-life of antibodies in the plasma [21, 36].

Although large-scale randomized clinical trials will ultimately confirm the safety and efficacy of convalescent plasma therapy for COVID-19, a recent safety study by Joyner and colleagues [12] provided encouraging data. By analyzing key safety metrics following transfusion of convalescent plasma in 5,000 hospitalized adults with severe or life-threatening COVID-19, the incidence of serious adverse events was found to be <1%, and the 7-day mortality incidence was 14.9%. These early indicators are encouraging and highly supportive of the use of convalescent plasma as a rescue therapy in hospitalized patients, given that the reported fatality rate of COVID-19 among patients admitted to the intensive care units (ICUs) is 57% [37]. In fact, the low risk indicated by the study support expanding the use of convalescent plasma therapy in less ill individuals [6].

## Future perspectives

The global reach of the COVID-19 pandemic and the desperate need for effective treatments have provided an impetus to develop convalescent plasma therapy into a viable (albeit short-term) treatment option particularly for the critically ill. Although its efficacy and safety have not yet been fully proven, convalescent plasma therapy for COVID-19 patients is projected to be a safe and potentially effective therapy for prophylaxis and treatment. However, it is critically important to perform rigorous randomized controlled trials to confirm efficacy and safety and to provide evidence for improved meaningful clinical outcomes. Nevertheless, despite the nuanced challenges, the substantial evidence of benefit with use for prior viral infections offers strong precedent for convalescent plasma as a therapeutic approach. Importantly, efforts should be focused not only on evaluation of the feasibility of plasma treatment for infectious diseases but also ensure that use of convalescent plasma therapy takes place according to ethical and controlled conditions.
